# Comparison of visceral fat measurement by dual-energy X-ray absorptiometry to computed tomography in HIV and non-HIV

**DOI:** 10.1038/s41387-019-0073-1

**Published:** 2019-02-25

**Authors:** Lindsay T. Fourman, Emma M. Kileel, Jane Hubbard, Tara Holmes, Ellen J. Anderson, Sara E. Looby, Kathleen V. Fitch, Meghan N. Feldpausch, Martin Torriani, Janet Lo, Takara L. Stanley, Steven K. Grinspoon

**Affiliations:** 1 Program in Nutritional Metabolism, Department of Medicine, Massachusetts General Hospital, Boston, MA USA; 2 Translational and Clinical Research Center, Massachusetts General Hospital, Boston, MA USA; 3 Yvonne L. Munn Center for Nursing Research, Massachusetts General Hospital, Boston, MA USA; 4 MGH Metabolic Imaging Core, Department of Radiology, Massachusetts General Hospital, Boston, MA USA

## Abstract

**Background/Objectives:**

Individuals with HIV are susceptible to visceral fat accumulation, which confers an increased risk of cardiometabolic disease. Advanced software to ascertain visceral fat content from dual-energy X-ray absorptiometry (DXA) has not been validated among this population. We sought to compare DXA with computed tomography (CT) in the measurement of visceral fat cross-sectional area (VAT) in HIV and non-HIV using Bland–Altman analyses.

**Subjects/Methods:**

Data were combined from five previously conducted studies of individuals with HIV (*n* = 313) and controls without HIV (*n* = 144) in which paired DXA and CT scans were available. In cross-sectional analyses, DXA-VAT was compared with CT-VAT among participants with and without HIV. In longitudinal analyses, changes in VAT over time were compared between DXA and CT among participants with and without HIV receiving no intervention over 12 months and among individuals with HIV receiving tesamorelin—a medication known to reduce VAT—over 6 months.

**Results:**

In HIV, DXA underestimated VAT compared with CT among individuals with increased visceral adiposity. The measurement bias was −9 ± 47 cm^2^ overall, but became progressively larger with greater VAT (*P* < 0.0001), e.g., −61 ± 58 cm^2^ among those with VAT ≥ 200 cm^2^. Sex-stratified analyses revealed that the relationship between VAT and measurement bias was especially pronounced in men (*P* < 0.0001). Longitudinally, DXA underestimated changes in VAT, particularly among those at the extremes of VAT gain or loss (*P* < 0.0001). In contrast to the cross-sectional findings, the tendency for DXA to underestimate longitudinal changes in VAT was evident in both men and women. Analogous findings were seen among controls in cross-sectional and longitudinal analyses.

**Conclusions:**

DXA underestimated VAT relative to CT in men with and without HIV, who had increased visceral adiposity. DXA also underestimated changes in VAT over time in men and women, irrespective of HIV status. DXA-VAT should be used with caution among both HIV and non-HIV-infected populations.

## Introduction

Independent of body mass index (BMI), fat distribution is an important determinant of cardiometabolic health^1^. Specifically, visceral fat has been associated with hypertension^2,3^, dyslipidemia^3,4^, glucose intolerance^3,4^, and coronary artery disease^5,6^. On the other hand, subcutaneous fat has been generally regarded as a benign storage depot^3,4,7^. Among individuals with HIV, visceral fat accumulation is commonly observed, even at a normal or overweight BMI^8,9^. Moreover, consonant with findings from the general population, HIV-related changes in fat distribution have been shown to confer an increased risk of cardiovascular disease^10–12^.

In HIV and in the general population, there is a critical need for a safe, affordable, and accurate method to quantify visceral fat. Such an approach would allow for the routine clinical use of visceral fat measurement to stratify cardiovascular risk and to monitor the efficacy of metabolic interventions. The gold standard for abdominal adipose tissue measurement is computed tomography (CT)^13^. In this regard, visceral fat cross-sectional area at the L4 vertebral level (CT-VAT) has been widely adopted due to its strict relation to skeletal landmarks and thus high degree of reproducibility (*r* = 0.99)^13,14^. However, as CT involves radiation exposure and is costly, alternative means to assess visceral fat have been sought^15^.

Dual-energy X-ray absorptiometry (DXA) is an inexpensive imaging modality that uses minimal radiation to quantify regional fat distribution (e.g., total abdominal or truncal fat)^16–18^. In recent years, advanced software has enabled visceral fat cross-sectional area to be ascertained in a predominantly automated manner from a standard DXA scan (DXA-VAT)^19^. Multiple studies among the general population have demonstrated that DXA-VAT approximates VAT as measured by other imaging modalities including CT^15,19–21^. However, the accuracy of DXA-VAT has not been evaluated among individuals with HIV, who may exhibit substantial expansion of this depot. Moreover, the use of DXA to quantify changes in visceral fat over time (i.e., in response to an intervention) has never before been assessed among any patient population.

In the current analysis, we leveraged data from previously conducted clinical studies to compare DXA-VAT with CT-VAT among a large sample of men and women with and without HIV. Specifically, we sought to assess the accuracy of DXA in the measurement of (1) visceral fat at a single time point, (2) changes in visceral fat over time that occur spontaneously, and (3) changes in visceral fat over time that occur in response to an intervention. Before DXA-VAT can be readily applied in clinical and research settings, it is pivotal to validate its accuracy in diverse populations and to delineate potential limitations to its use.

## Subjects and methods

### Subjects

We combined all available data from five studies of individuals with HIV, which were conducted from 2006 to 2014 (Supplementary Table 1)^22–26^. In each study, participants recruited from the Boston area underwent abdominal CT and DXA concurrently per protocol. Controls without HIV were co-enrolled in three of the studies and their data were included for comparison^22,23,25^. In four of the studies, participants were followed longitudinally for at least 6 months with CT and DXA repeated at fixed intervals^23–26^.

In our cross-sectional analysis, we compared DXA with CT in the measurement of visceral fat among individuals with and without HIV at a single time point. Each participant contributed one set of paired CT and DXA data to this analysis (Supplementary Fig. 1). For subjects who took part in more than one study, only baseline data from their first longitudinal study was included for analysis. If no longitudinal data was available, data from the cross-sectional study was used. We prioritized inclusion of baseline longitudinal data in the cross-sectional analysis to facilitate consistency between the cross-sectional and longitudinal sub-studies.

In our longitudinal analysis, we sought to determine the accuracy of DXA compared with CT with respect to repeated measurements of visceral fat over time. This analysis consisted of two arms (Supplementary Fig. 1). First, we devised a natural history group, which comprised participants with or without HIV, who had been assigned to receive either no treatment or placebo for at least 12 months^23,25,26^. Changes in visceral fat from baseline to 12 months as measured by DXA and CT were compared. As with our cross-sectional analysis, for subjects who had participated in more than one longitudinal study, only data from their first study was eligible for inclusion. Second, we assembled a group of individuals with HIV, who had been randomized to receive tesamorelin or placebo for 6 months^24^. Tesamorelin is a growth hormone-releasing hormone (GHRH) analog that selectively reduces visceral fat without substantially altering subcutaneous fat content or BMI^27,28^. As such, this randomized trial enabled us to assess the agreement between DXA and CT with regard to an intervention known to reduce visceral fat. We included all participants of this study, who had DXA and CT data available at baseline and 6 months.

Subjects gave informed consent before their participation in study procedures. The Institutional Review Boards at Massachusetts General Hospital and/or Massachusetts Institute of Technology approved each original study and the current combined analysis.

### Study procedures

In each of the five studies, all participants underwent a detailed history and physical exam. HIV viral load and T-cell subsets were performed using standard assays. At baseline, whole-body DXA (Hologic QDR 4500 A, Hologic Discovery A, and Hologic Horizon A) was performed and analyzed by licensed research dieticians certified as bone densitometry technologists by the International Society for Clinical Densitometry. In addition, at baseline, each subject underwent a single-slice CT scan of the abdomen at L4 level (General Electric, Waukesha, WI) for assessment of VAT and subcutaneous adipose tissue area (SAT). In four of the five studies, repeated DXA and CT were performed at set intervals of 6 or more months among participants receiving either treatment, placebo, or no intervention.

CT scan was performed with participants in the supine position. Lateral and frontal scout images were obtained to identify the L4 vertebral level, which served as a landmark for the single-slice scan. Scan parameters were standardized: 144 table height, 80 kV, 70 mA, 2 seconds scan time, 1 cm slice thickness, 48 cm field of view. A single expert (M.T.) supervised the reading of all scans to ensure uniformity. Quantification of abdominal fat depots was performed using image analysis software (ViTrak, Merge/eFilm, Inc., Chicago, IL). Briefly, thresholding methods were applied to identify adipose tissue using a threshold set to −50 to −250 Hounsfield units. Then, manual delineation with tools provided by the software was used to separate VAT from SAT. Pixels within the threshold that were not anatomically one of the two adipose tissue depots were removed. Using this technique, the coefficient of variation for repeated measurements of the same scan on consecutive days by the same analyst has been reported as 2.3% for VAT and 1.7% for SAT^20^.

For the purposes of the current analysis, DXA was re-analyzed by a single research dietician (J.H.) in 2018 using Hologic Horizon A, APEX 6.6.0.5 software (Hologic, Inc., Bedford, MA, USA) for measurement of visceral fat cross-sectional area. Standard procedures per the Horizon Bone Densitometry Systems User Guide were employed^29^. Briefly, for each scan, the software automatically located the outer and inner margins of the abdominal wall in a 5 cm region of the abdomen. The lower border of this region coincided with the L4 vertebral level and marked the plane through which DXA-VAT cross-sectional area was reported. The software measured total abdominal fat and subcutaneous fat directly, and reported visceral fat content as the difference between these measurements^20,30^. To assess the reproducibility of our DXA-VAT readings, 98 DXA scans were re-analyzed by a second research dietician (T.H.). Owing to the high level of automaticity of the APEX software, there was a strong correlation (*r* > 0.99) of DXA-VAT measurements between readers. Both dieticians were kept blind to the CT data.

### Statistical analysis

In our comparisons of DXA vs. CT, individuals with and without HIV were analyzed separately. For the cross-sectional analysis, the correlation between CT-VAT and DXA-VAT was determined using Pearson’s correlation coefficient. Measurement bias was calculated as the mean difference between the two techniques (DXA–CT) ± SD. Bland–Altman plots were subsequently used to display the relationship of average VAT between modalities vs. measurement bias^31^. Visual inspection followed by linear regression was used to assess for systematic bias. Multivariable models also were constructed to adjust for age, sex, race, and ethnicity, and to test for interactions of VAT with age (≥ vs. <50 years), sex, race, BMI (≥ vs. <30 kg/m^2^), CD4 count (≥ vs. <250 cells/mm^3^), HIV viral load (≥ vs. <50 copies/mL), and antiretroviral therapy (ART) duration (≥ vs. <5 years), and class (among individuals receiving ART). For interactions that were significant, subgroup analyses were performed. A comparable statistical approach was used to compare DXA with CT with respect to SAT.

For the natural history longitudinal analysis, a linear regression was performed to relate change in CT-VAT to change in DXA-VAT over 12 months. Multivariable models were constructed to compare the changes in VAT between these two modalities, while controlling for age, sex, race, and ethnicity, and to test for an interaction between sex and change in VAT. Changes in VAT as measured by DXA and CT also were compared using a Bland–Atlman plot. An analogous statistical approach was taken for the tesamorelin longitudinal analysis, which compared changes in visceral fat as measured by CT and DXA over 6 months among individuals with HIV only.

All two-group comparisons used Student’s two-tailed *t*-test assuming unequal variances for continuous outcomes and *χ*^2^-test for categorical outcomes. Continuous variables were reported as mean ± SD or mean (95% confidence interval). A critical value of *P* < 0.05 was used to designate statistical significance. All statistical analyses were performed using JMP Pro 12.0.1 (SAS Institute, Inc., Cary, NC).

## Results

### Clinical characteristics

A total of 313 individuals with HIV and 144 controls were included in these analyses (Table 1). Participants with and without HIV were similar in age (48 ± 7 vs. 47 ± 7 years) and racial distribution. Individuals with HIV tended to have long-standing infection (15 ± 7 years) that was well controlled (viral load < 50 copies/mL in 76%) on ART (88%).

**Table 1:** Baseline characteristics in HIV and non-HIV groups

	HIV (*n* = 313)	Non-HIV (*n* = 144)	*P*-value
Demographic characteristics			
Age, years	48 ± 7	47 ± 7	0.25
Male, %	66	42	<0.0001
Race, %			0.15
White	56	51	
Black	35	43	
Asian	1	2	
American Indian	1	1	
Other	7	3	
Ethnicity, %			
Hispanic	13	6	0.03
Body composition characteristics			
BMI, kg/m^2^	27 ± 5	28 ± 5	0.09
BMI category, %			0.55
Overweight	40	44	
Obese	26	26	
Waist iliac, cm	97 ± 14	97 ± 15	0.69
CT-TAT, cm^2^	378 ± 179	404 ± 200	0.19
CT-VAT, cm^2^	141 ± 101	115 ± 84	0.004
CT-SAT, cm^2^	236 ± 137	289 ± 152	0.0005
HIV-related characteristics			
Duration of HIV, years	15 ± 7		
CD4 T lymphocytes, cells/mm^3^	583 ± 298		
CD8 T lymphocytes, cells/mm^3^	864 ± 469		
Viral load < 50 copies/mL, %	76		
Current ART, %	88		
Duration of ART, years	7 ± 5		
Current NRTI, %	85		
Duration of NRTI, years	6 ± 5		
Current NNRTI, %	32		
Duration of NNRTI, years	2 ± 4		
Current PI, %	47		
Duration of PI, years	3 ± 4		

Demographic, body composition, and HIV-related characteristics are depicted for the HIV and non-HIV groups. Continuous variables are shown as mean ± SD. Categorical variables are shown as frequency (%). *ART* antiretroviral therapy, *NNRTI* non-nucleoside reverse transcriptase inhibitor, *NRTI* nucleoside reverse transcriptase inhibitor, *PI* protease inhibitor, *SAT* subcutaneous adipose tissue cross-sectional area, *VAT* visceral adipose tissue cross-sectional area

*P*-value for the difference between HIV and non-HIV groups was determined with Student’s *t*-test (continuous data) or *χ*^2^-test (categorical data)

Although BMI was comparable between HIV and non-HIV groups (27 ± 5 vs. 28 ± 5 kg/m^2^), fat distribution varied as would be expected^8,32^. In particular, individuals with HIV had higher CT-VAT (141 ± 101 vs. 115 ± 84 cm^2^, *P* = 0.004) and lower CT-SAT (236 ± 137 vs. 289 ± 152 cm^2^, *P* = 0.0005) than controls. Total abdominal adiposity did not differ between groups.

### Cross-sectional comparison of DXA with CT in HIV

**Fig. 1:**
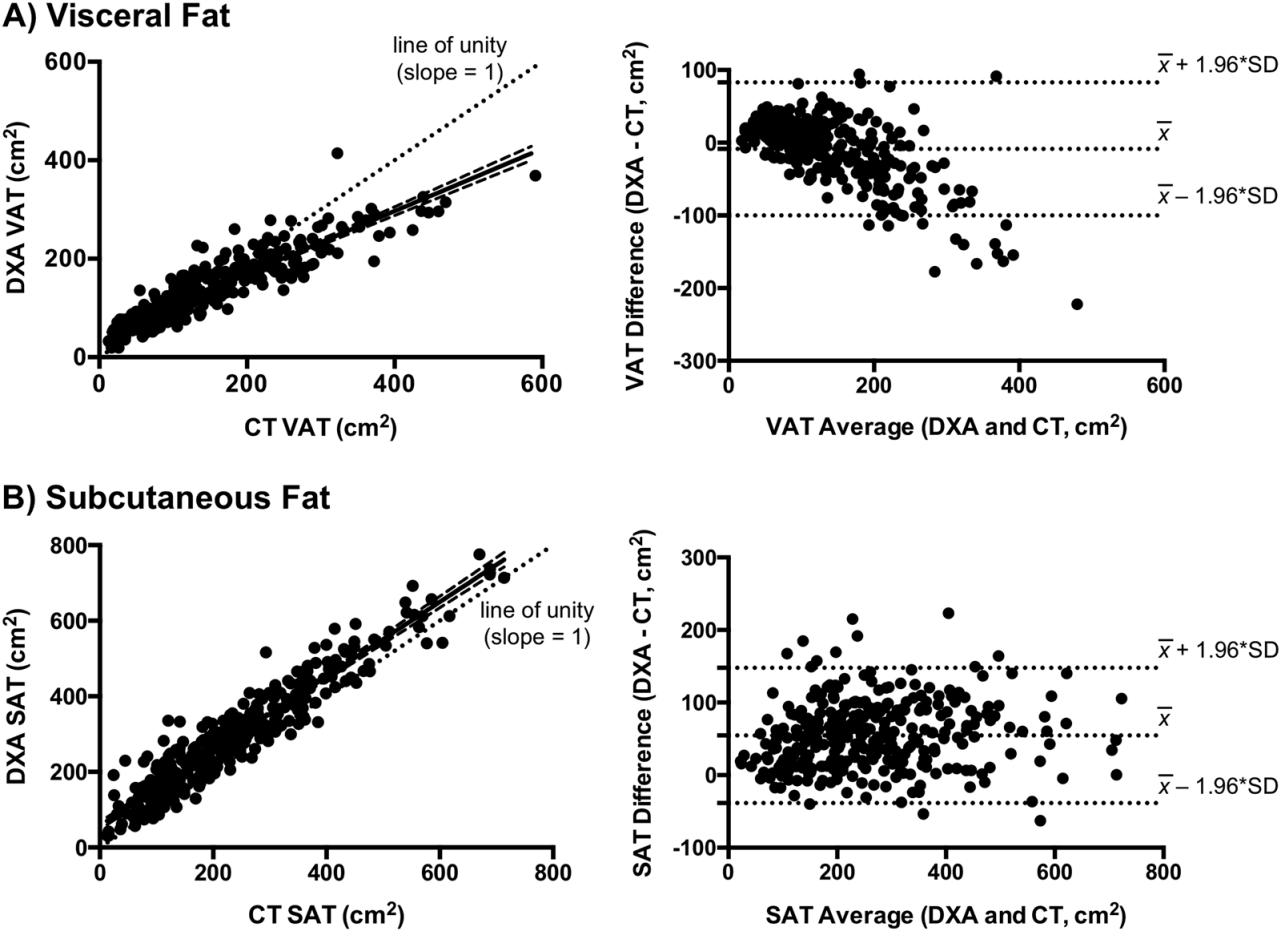
Comparison of DXA and CT for cross-sectional VAT and SAT measurement in HIV. **a** In HIV, there is a strong correlation between CT and DXA in the measurement of VAT (*r* = 0.91, *P* < 0.0001), although the regression line (shown with 95% confidence bands) deviates from the dotted line of unity (*P* < 0.05). The Bland–Altman plot for VAT demonstrates that measurement bias (DXA–CT) is progressively more negative with greater visceral fat content (*P* < 0.0001). Horizontal dotted lines denote the mean difference between DXA and CT, as well as the expected 95% limits of agreement. Measurement bias in individuals with the most severe visceral fat accumulation is not contained within the scope of these lines. **b** There is a strong correlation between CT and DXA in the measurement of SAT (*r* = 0.94, *P* < 0.0001). Unlike VAT, the regression line (shown with 95% confidence bands) does not differ from the dotted line of unity. The Bland–Altman plot for SAT demonstrates that DXA overestimates SAT consistently across the subcutaneous fat spectrum. Horizontal dotted lines denote the mean difference between DXA and CT, as well as the expected 95% limits of agreement

Among individuals with HIV, CT-VAT and DXA-VAT were strongly correlated (*r* = 0.91, *P* < 0.0001) (Fig. 1a). Moreover, DXA-VAT only mildly underestimated CT-VAT on average (−9 ± 47 cm^2^, *P* = 0.001) (Table 2). However, the difference between these modalities (DXA–CT) became progressively more negative with greater visceral fat content (*P* < 0.0001) (Fig. 1a). As such, the measurement bias was 7 ± 29 cm^2^ among individuals with VAT < 200 cm^2^, compared with −61 ± 58 cm^2^ among those with VAT ≥ 200 cm^2^ (*P* < 0.0001).

**Table 2:** Comparison of visceral fat area measured by DXA and CT

	*n*	DXA-VAT, cm^2^	CT-VAT, cm^2^	VAT difference (DXA–CT), cm^2^
HIV	313	133 ± 70	141 ± 101	−9 ± 47
Women	106	110 ± 59	96 ± 64	14 ± 27
Men	207	145 ± 72	165 ± 108	−20 ± 50
Non-HIV	144	116 ± 63	115 ± 84	1 ± 40
Women	83	111 ± 63	96 ± 69	15 ± 29
Men	61	122 ± 62	141 ± 95	−19 ± 44

VAT as measured by DEXA and CT, as well as the measurement difference between modalities, are presented as mean ±SD. *CT* computed tomography, *DXA* dual-energy X-ray absorptiometry, *VAT* visceral fat cross-sectional area

**Fig. 2:**
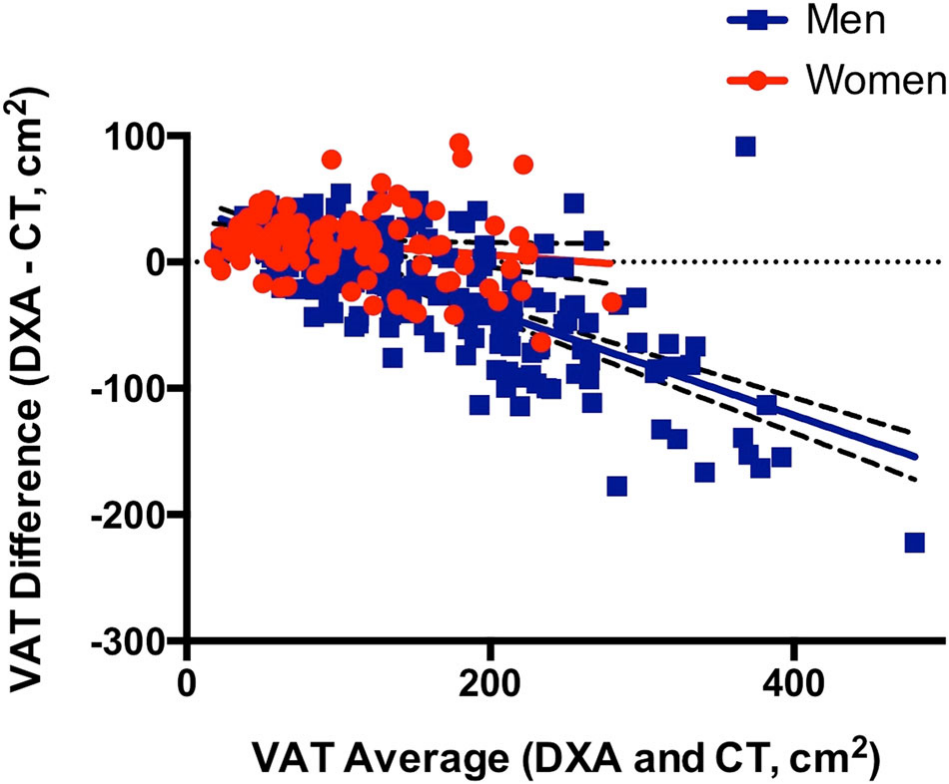
Sex differences in VAT measurement bias in HIV Sex-stratified analyses reveal that the inverse relationship between visceral adiposity and measurement bias (DXA–CT) in HIV is predominantly driven by men (*P* < 0.0001). In the Bland–Altman plot shown, the line for men (blue squares) has a slope of −0.41 (−0.47 to −0.36), whereas the line for women (red circles) has a slope of −0.09 (−0.17 to −0.003). Accordingly, DXA most substantially underestimates VAT among HIV-infected men with visceral fat accumulation. Linear regression lines are shown with 95% confidence bands

Sex-stratified analyses revealed contrasting relationships of DXA-VAT with CT-VAT in men vs. women. Clinical characteristics between men and women with HIV were compared in Supplementary Table 2. On average, DXA-VAT underestimated CT-VAT among men, whereas it overestimated CT-VAT among women (−20 ± 50 vs. 14 ± 27 cm^2^, *P* < 0.0001) (Table 2). The relationship between visceral adiposity and measurement bias (DXA–CT) also differed between sexes as evidenced by a significant sex × VAT interaction (*P* < 0.0001) in models that adjusted for age, race, and ethnicity. When each sex was analyzed separately, the line relating VAT average (DXA and CT) to VAT difference (DXA–CT) had a slope of −0.41 (−0.47 to −0.36) in men and −0.09 (−0.17 to −0.003) in women (Fig. 2). Thus, DXA progressively underestimated VAT as a function of visceral fat content in men, whereas this relationship was less pronounced in women.

In addition, in HIV, obesity did not modify the inverse relationship between visceral adiposity and measurement bias (DXA–CT). However, at any given visceral fat content, the measurement bias was modestly smaller in obese compared with non-obese individuals (Supplementary Fig. 2). There was no effect or interaction with VAT for age, race, CD4 count, HIV viral load, or ART duration or class (among participants receiving ART) in relation to measurement bias.

**Fig. 3:**
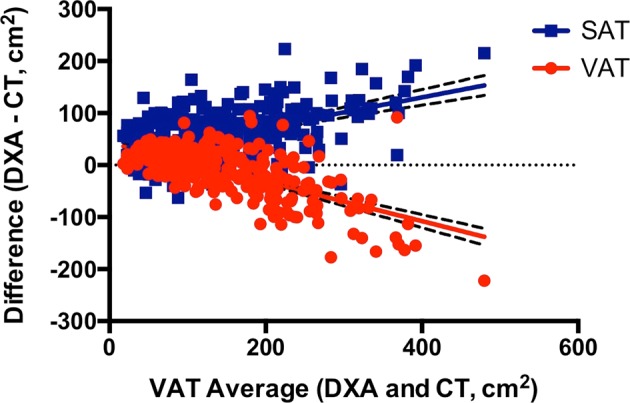
Reciprocal relationship between VAT and SAT measurement bias in HIV Among HIV-infected individuals, DXA progressively underestimates VAT (red circles) in proportion to visceral fat accumulation (*P* < 0.0001). Conversely, DXA increasingly overestimates SAT (blue squares) as a function of visceral adiposity (*P* < 0.0001). Linear regression lines are shown with 95% confidence bands

In HIV, CT-SAT and DXA-SAT were strongly correlated (*r* = 0.94, *P* < 0.0001) (Fig. 1b) with an overall measurement bias (DXA–CT) of 55 ± 48 cm^2^ (*P* < 0.0001). Unlike visceral fat, the bias did not substantially vary across the subcutaneous fat spectrum (Fig. 1b). However, among individuals with high visceral fat in whom DXA routinely underestimated VAT, DXA conversely overestimated SAT (Fig. 3). Thus, SAT bias (DXA–CT) became increasingly more positive as a function of visceral adiposity (*P* < 0.0001), although it did not systematically relate to subcutaneous fat content per se.

### Longitudinal comparison of DXA with CT in HIV

**Fig. 4:**
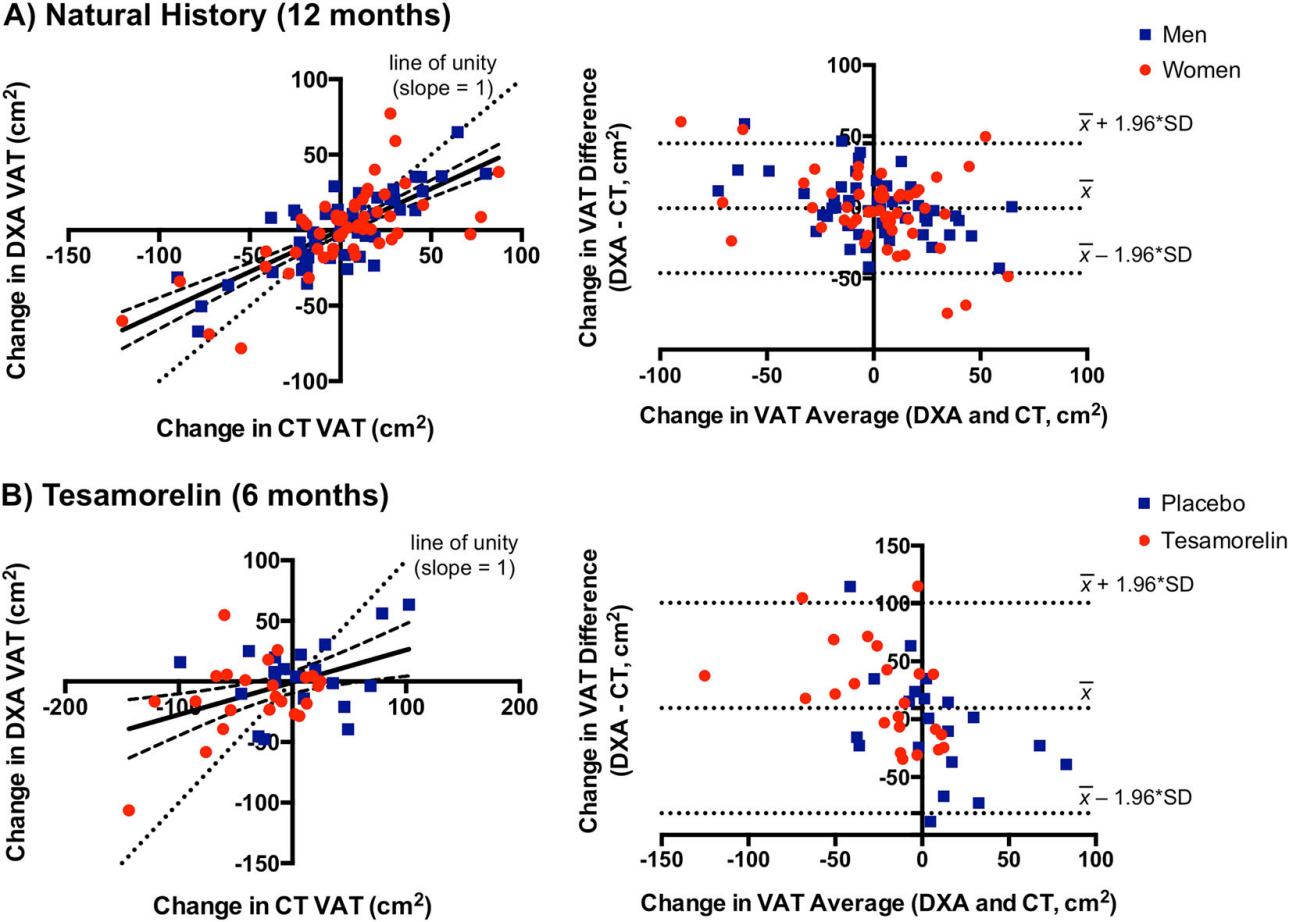
Comparison of DXA and CT for longitudinal VAT measurement in HIV. **a** In the natural history analysis of individuals with HIV, changes in VAT over 12 months by CT and DXA are well-correlated (*r* = 0.74, *P* < 0.0001*)*. However, the regression line (shown with 95% confidence bands) deviates from the dotted line of unity (*P* < 0.05) such that DXA underestimates a gain or loss of VAT as measured by CT. The Bland–Altman plot comparing changes in VAT over 12 months between DXA and CT is also shown. Measurement bias (DXA–CT) is positive among individuals with VAT loss and negative among individuals with VAT gain (*P* < 0.0001), which again suggests that VAT gain and loss are both underestimated by DXA. Horizontal dotted lines denote the mean difference between DXA and CT in change in VAT over time and the expected 95% limits of agreement. Measurement bias between DXA and CT does not differ between men (blue squares) and women (red circles). **b** The tesamorelin analysis similarly shows that DXA underestimates changes in VAT over 6 months relative to CT. The extent of underestimation is comparable between individuals treated with tesamorelin (red circles) or placebo (blue squares)

A total of 106 individuals with HIV who were followed for at least 12 months on no active treatment were included in the natural history analysis. The linear relationship between change in CT-VAT and change in DXA-VAT over 12 months had a slope of 0.55 (0.45–0.65), indicating that DXA systematically underestimated CT with respect to change in VAT over time (Fig. 4a). As such, among the subset of participants who had a decline in CT-VAT at 12 months compared with baseline (*n* = 46), change in CT-VAT was −27 ± 28 cm^2^, whereas change in DXA-VAT was −15 ± 24 cm^2^. Similarly, among the subset of participants who had a gain in CT-VAT over 12 months (*n* = 60), change in CT-VAT was 23 ± 21 cm^2^, compared with change in DXA-VAT of 13 ± 20 cm^2^. Unlike the sex differences seen in the cross-sectional analysis, sex did not modify the relationship between change in CT-VAT and change in DXA-VAT over 12 months in a model that adjusted for age, race, and ethnicity. The tendency for DXA to underestimate changes in VAT also was evident in a Bland–Altman plot, in which the measurement bias was largest at the most extreme gains and losses of VAT (Fig. 4a). In this regard, measurement bias (DXA–CT) was negative among individuals who had a gain in VAT and positive among individuals who had a loss of VAT (*P* < 0.0001).

We next examined subjects with HIV, who had been assigned to receive tesamorelin (*n* = 23) or placebo (*n* = 20) for 6 months in a randomized-controlled trial. Analogous to the natural history analysis, among the tesamorelin-treated participants, the linear relationship between change in CT-VAT and change in DXA-VAT had a slope that was significantly <1 (0.29, 0.01–0.57). In this group, over 6 months, the change in CT-VAT was −33 ± 46 cm^2^, whereas the change in DXA-VAT was −12 ± 31 cm^2^. When the placebo-treated participants from the original randomized-controlled trial were included in the model, treatment assignment did not modify the relationship between change in CT-VAT and change in DXA-VAT over time (Fig. 4b). The Bland–Altman plot of the data from the tesamorelin trial was comparable to that seen in the natural history analysis (Fig. 4b).

### Comparison of DXA to CT in controls without HIV

The comparison between DXA and CT in controls without HIV yielded similar findings to those seen in HIV. There was a strong correlation between CT-VAT and DXA-VAT (*r* *=* 0.89, *P* < 0.0001) (Supplementary Fig. 3) and only a modest bias of DXA compared with CT overall (1 ± 40 cm^2^) (Table 2). However, as in HIV, the measurement difference between modalities (DXA–CT) became progressively more negative with greater visceral adiposity (*P* < 0.0001) (Supplementary Fig. 3). In addition, once again, men predominantly drove this relationship with a significant sex × VAT interaction in a model that adjusted for age, race, and ethnicity (*P* < 0.0001) (Supplementary Fig. 4). Lastly, although there was no obesity × VAT interaction, the magnitude of the measurement bias was slightly smaller in obese compared with non-obese individuals across the spectrum of visceral adiposity (Supplementary Fig. 2).

A total of 80 controls without HIV were included in the longitudinal analysis. As in HIV, the linear regression relating change in CT-VAT to change in DXA-VAT over 12 months had a slope that was <1 (0.51, 0.39–0.62), suggesting that DXA underestimated longitudinal changes in VAT compared with CT (Supplementary Figure 5). This relationship was not modified by sex in a model that adjusted for age, race, and ethnicity.

## Discussion

In the current work, we used data from five previously conducted studies of participants with and without HIV, to assess the accuracy of DXA in the automated measurement of VAT. In our cross-sectional analysis of individuals with HIV, we showed that DXA underestimated VAT relative to CT among those with high visceral fat content. Sex-stratified comparisons revealed this measurement bias was largely driven by men rather than women, even upon controlling for VAT. In our longitudinal assessment of participants with HIV, we found that DXA underestimated changes in VAT over time, irrespective of sex. Analogous findings were demonstrated in cross-sectional and longitudinal comparisons among controls without HIV.

To our knowledge, this is the first study to examine the accuracy of DXA-VAT specifically among individuals with HIV. People living with HIV as a group are prone to visceral fat accumulation due to an interplay of medication toxicities, viral effects on host metabolism, and contemporary lifestyle trends^33,34^. Moreover, HIV lipodystrophy is an extreme form of visceral fat accumulation that remains common among long-term survivors^35^ or those with drug resistance, who require older-generation therapies^36^. Visceral fat accumulation is a key mediator of cardiometabolic comorbidities in HIV such as glucose intolerance^12,37^, nonalcoholic fatty liver disease^38^, and coronary artery disease^39^. Accordingly, our group developed the GHRH analog tesamorelin as a novel strategy to selectively reduce visceral fat in HIV without substantially altering subcutaneous fat or BMI^27,28^. As anticipated, a reduction in visceral fat with tesamorelin has been associated with metabolic benefits including decreased triglycerides^40^ and liver enzymes^41^ among this patient population. Tesamorelin is the only medication that has been approved by the Food and Drug Administration to treat excess abdominal fat in HIV.

Given the pivotal contribution of visceral fat accumulation to cardiometabolic risk in HIV, a technique that allows for the safe, affordable, and convenient measurement of visceral fat may substantially improve the quality of care among the HIV population. Despite the initial promise of DXA in this regard, we found that this modality systematically underestimated VAT among participants with HIV and visceral fat accumulation compared with CT. Of note, sex-specific comparisons revealed that men rather than women were predominantly responsible for this relationship. In contrast to VAT, the measurement difference for SAT between DXA and CT did not substantially vary across the subcutaneous fat spectrum. However, for the individuals in whom DXA underestimated VAT, DXA conversely overestimated SAT. Our findings were analogous for controls without HIV, although the range of visceral fat content examined was narrower among this group.

Multiple studies from the general population have posited that visceral fat quantification by DXA is accurate compared with CT and magnetic resonance imaging (MRI)^15,19–21,42^. However, in several of these reports, the sample was limited to women^20,21^. Concordant with these studies, our data suggest that DXA is relatively accurate in women with and without HIV, even among those with increased visceral adiposity.

Moreover, consonant with our findings in men with and without HIV, several previous studies have shown that DXA loses accuracy with greater visceral adiposity^15,42,43^. In addition, multiple prior reports have found a tendency for DXA to underestimate visceral fat compared with other modalities^43,44^. In one such study of older males, visceral fat volume as measured by DXA was consistently 30% less than that measured by MRI^44^. Of note, the authors of this previous work attributed this bias to a difference in compartment sizes between modalities rather than to a true measurement inaccuracy^44^. Similarly, a study among Korean men and women found that DXA underestimated visceral fat volume relative to CT as a function of visceral adiposity^43^. Sex-stratified analyses were not performed in this previous study^43^. A higher proportion of visceral fat is common to men^45^ and Asians^46^ relative to women and other races, respectively, which may account for the more pronounced measurement bias seen among these groups.

Strategies to reduce visceral fat have been developed for the clinical care of individuals with HIV as a means toward improving metabolic health. However, to our knowledge, the accuracy of DXA with respect to detecting changes in visceral fat over time has never before been assessed. In the current analysis, among individuals with and without HIV receiving no intervention for 12 months, we found that DXA systematically underestimated changes in VAT as measured by CT, irrespective of sex. We additionally showed that DXA underestimated changes in VAT over 6 months among individuals with HIV that were randomized to receive tesamorelin or placebo. Among the tesamorelin-treated participants, the average VAT reduction as measured by DXA was <50% of that measured by CT.

Strengths of this analysis include its large sample of individuals with and without HIV. Furthermore, both men and women were included in our analysis, which allowed for us to draw important sex-specific distinctions between groups. In addition, we assessed longitudinal changes in DXA-VAT over time both in the presence and the absence of a metabolic intervention. An important limitation to our analysis is that DXA-VAT was exclusively determined using a Hologic scanner and APEX software. Although this lends consistency to our comparisons, we were not able to assess the accuracy of DXA-VAT as ascertained by other systems. Similarly, our analyses compared DXA and CT with respect to visceral fat cross-sectional area, but did not evaluate visceral fat volume or mass. Measurement of visceral fat volume by CT was not performed in our original studies to minimize the dose of radiation to which participants were exposed. Nonetheless, as visceral fat cross-sectional area is highly correlated with visceral fat volume on CT (*r* > 0.95)^47^, we would expect a comparison of this parameter between modalities to yield similar findings. However, further research is needed to confirm this.

In the current study, we showed that DXA underestimated visceral fat content among men with and without HIV, who had increased visceral adiposity. We also demonstrated that DXA underestimated changes in visceral fat content over time among both men and women, irrespective of HIV status. These findings represent important caveats to the use of DXA for the automated determination of visceral fat. DXA-VAT should be interpreted with caution in HIV, particularly among men.

## Supplementary information

Supplemental Figure Legends

Supplemental Table 1

Supplemental Table 2

Supplemental Figure 1

Supplemental Figure 2

Supplemental Figure 3

Supplemental Figure 4

Supplemental Figure 5
