# Supplementary material for: Comparison of visceral fat measurement by dual-energy X-ray absorptiometry to computed tomography in HIV and non-HIV
Source: Nutr Diabetes. 2019 Feb 25;9:6. doi: 10.1038/s41387-019-0073-1 (PMC6389911; doi:10.1038/s41387-019-0073-1)
Supplement: Supplementary file 1 — Supplemental Figure Legends [file 41387_2019_73_MOESM1_ESM.pdf]

Supplemental Figure 1: Study Schema. DXA and CT were compared with respect to VAT measurement in cross-sectional and longitudinal analyses among individuals with and without HIV as shown.

Supplemental Figure 2: VAT Measurement Bias in Individuals with and without Obesity. A) In HIV, the inverse relationship between visceral adiposity and measurement bias (DXA – CT) was similar between obese (purple squares) and non-obese (green circles) individuals. However, this difference between modalities was slightly smaller in magnitude among obese relative to non-obese participants (+15 cm^2, 10 to 20 cm^2, $P < 0.0001$). **B)** Similarly, in non-HIV, there was no VAT × obesity interaction, although obese individuals had a slightly smaller measurement bias at any given VAT (+9 cm^2, 2 to 16 cm^2, $P = 0.01$). In both panels, linear regression lines with 95% confidence bands are shown.

Supplemental Figure 3: Comparison of DXA and CT for Cross-Sectional VAT and SAT Measurement in Non-HIV. A) Among individuals without HIV, CT-VAT and DXA-VAT are strongly correlated ($r = 0.89$, $P < 0.0001$). However, as in HIV, the linear regression line (shown with 95% confidence bands) deviates from the dotted line of unity ($P < 0.05$). The Bland-Altman plot for VAT demonstrates that DXA underestimates VAT in relation to CT as a function of visceral adiposity ($P < 0.0001$). Horizontal dotted lines denote the mean difference between DXA and CT as well as the expected 95% limits of agreement. **B)** Among individuals without HIV, CT-SAT and DXA-SAT are strongly correlated ($r = 0.97$, $P < 0.0001$), and the measurement bias does not change substantially with subcutaneous fat content.

Supplemental Figure 4: Sex Differences in VAT Measurement Bias in Non-HIV. As

in HIV, sex-stratified analyses among individuals without HIV demonstrate that the enlarging measurement bias with greater visceral adiposity is most pronounced in men ($P < 0.0001$). In the Bland-Altman plot shown, the line for men (blue squares) has a slope of -0.44 (-0.53 to -0.35), whereas the line for women (red circles) has a slope of -0.09 (-0.19 to 0.003). Linear regression lines are shown with 95% confidence bands.

Supplemental Figure 5: Comparison of DXA and CT for Longitudinal VAT Measurement in Non-HIV. As in HIV, among individuals without HIV observed over 12 months, changes in VAT as measured by CT and DXA are highly correlated ($r = 0.71$, $P < 0.0001$). However, the regression line (shown with 95% confidence bands) deviates from the dotted line of unity ($P < 0.05$) such that DXA underestimates a gain or loss of VAT as measured by CT. The Bland-Altman plot is also shown with horizontal dotted lines to denote the mean difference between DXA and CT in change in VAT over time as well as the expected 95% limits of agreement. As in HIV, measurement bias (DXA – CT) is positive among individuals with VAT loss and negative among individuals with VAT gain ($P < 0.0001$), which again indicates that VAT gain and loss are underestimated by DXA. This relationship does not differ between men (blue squares) and women (red circles).
