# Supplementary material for: Comparison of visceral fat measurement by dual-energy X-ray absorptiometry to computed tomography in HIV and non-HIV
Source: Nutr Diabetes. 2019 Feb 25;9:6. doi: 10.1038/s41387-019-0073-1 (PMC6389911; doi:10.1038/s41387-019-0073-1)
Supplement: Supplementary file 2 — Supplemental Table 1 [file 41387_2019_73_MOESM2_ESM.pdf]

Supplemental Table 1: Overview of Data Included in Current Analysis

Study	Purpose	HIV (M/F)	Non-HIV (M/F)	Design
1 [22]	To compare prevalence and characteristics of coronary atherosclerosis between individuals with and without HIV	93/39	36/23	Cross-Sectional
2 [23]	To assess rates and predictors of coronary plaque progression among individuals with and without HIV	43/29	25/27	Longitudinal Observational
3 [24]	To examine cardiometabolic effects of tesamorelin versus placebo among HIV-infected individuals with abdominal fat accumulation	38/4	0/0	Randomized Controlled Trial
4 [25]	To compare changes in metabolic and psychosocial indices between perimenopausal women with and without HIV	0/27	0/33	Longitudinal Observational
5 [26]	To investigate the effects of atorvastatin versus placebo on coronary plaque inflammation in HIV	33/7	0/0	Randomized Controlled Trial

Each study is listed with a reference to the main publication. Values reflect numbers of men and women included from each study in the cross-sectional analysis.
