# Supplementary material for: Comparison of visceral fat measurement by dual-energy X-ray absorptiometry to computed tomography in HIV and non-HIV
Source: Nutr Diabetes. 2019 Feb 25;9:6. doi: 10.1038/s41387-019-0073-1 (PMC6389911; doi:10.1038/s41387-019-0073-1)
Supplement: Supplementary file 3 — Supplemental Table 2 [file 41387_2019_73_MOESM3_ESM.pdf]

Supplemental Table 2: Baseline Characteristics of Men and Women with HIV

	Men (n = 207)	Women (n = 106)	*P*-value
**Demographic Characteristics**			
Age, years	48 ± 7	47 ± 8	0.39
Race, %			<0.0001
White	67	34	
Black	23	58	
Asian	1	0	
American Indian	0.5	2	
Other	8	6	
Ethnicity, %			
Hispanic	13	13	0.92
**Body Composition Characteristics**			
BMI, kg/m^2	27 ± 5	28 ± 6	0.04
Waist Iliac, cm	98 ± 13	96 ± 15	0.40
CT-TAT, cm^2	362 ± 172	410 ± 188	0.03
CT-VAT, cm^2	165 ± 108	96 ± 64	<0.0001
CT-SAT, cm^2	197 ± 112	315 ± 148	<0.0001
**HIV-Related Characteristics**			
Duration of HIV diagnosis, years	15 ± 7	14 ± 6	0.13
CD4 T lymphocytes, $cells/mm^3$	556 ± 289	641 ± 308	0.02
CD8 T lymphocytes, $cells/mm^3$	862 ± 485	869 ± 409	0.92
Viral Load < 50 copies/mL, %	74	81	0.17
Current ART, %	86	91	0.29
Duration of ART, years	7 ± 6	7 ± 5	0.87
Current NRTI, %	84	86	0.66
Duration of NRTI, years	7 ± 5	6 ± 5	0.69
Current NNRTI, %	37	22	0.005
Duration of NNRTI, years	2 ± 4	2 ± 3	0.33
Current PI, %	44	54	0.11
Duration of PI, years	4 ± 5	3 ± 4	0.70

Demographic, body composition, and HIV-related characteristics are depicted for the men and women with HIV. Continuous variables are shown as mean ± SD. Categorical variables are shown as frequency (%). ART, antiretroviral therapy; NNRTI, non-nucleoside reverse transcriptase inhibitor; NRTI, nucleoside reverse transcriptase inhibitor; PI, protease inhibitor; SAT, subcutaneous adipose tissue cross-sectional area; VAT, visceral adipose tissue cross-sectional area.

P-value for the difference between men and women with HIV was determined with Student's t-test (continuous data) or chi-square test (categorical data).
