# Supplementary figures and images for: Comparison of visceral fat measurement by dual-energy X-ray absorptiometry to computed tomography in HIV and non-HIV
Source: Nutr Diabetes. 2019 Feb 25;9:6. doi: 10.1038/s41387-019-0073-1 (PMC6389911; doi:10.1038/s41387-019-0073-1)

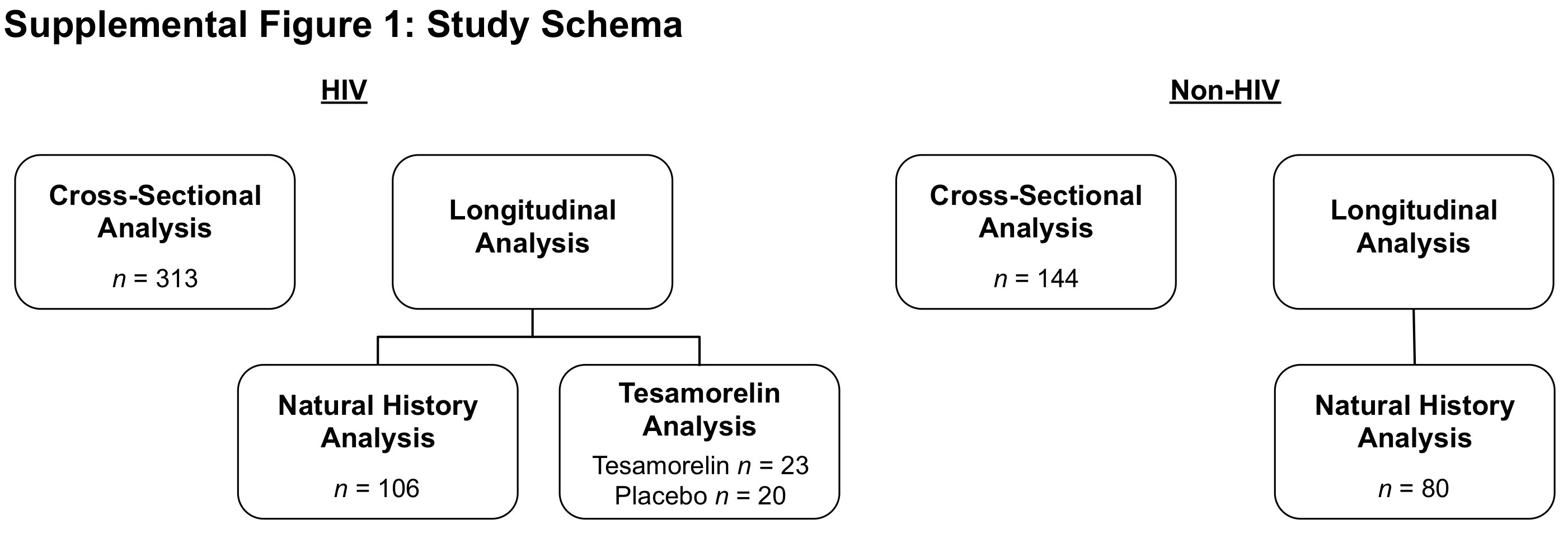

Supplement: Supplementary file 4 — Supplemental Figure 1 [file 41387_2019_73_MOESM4_ESM.jpg]

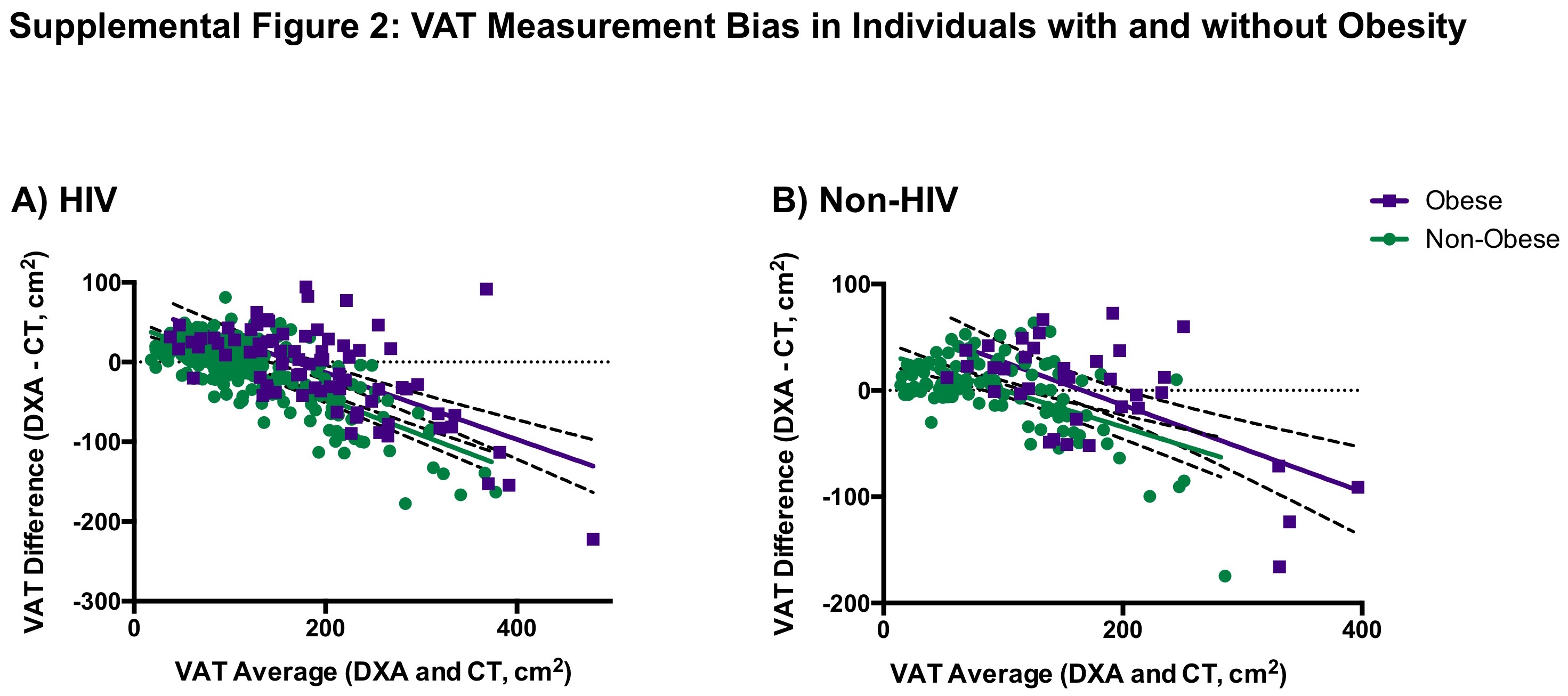

Supplement: Supplementary file 5 — Supplemental Figure 2 [file 41387_2019_73_MOESM5_ESM.jpg]

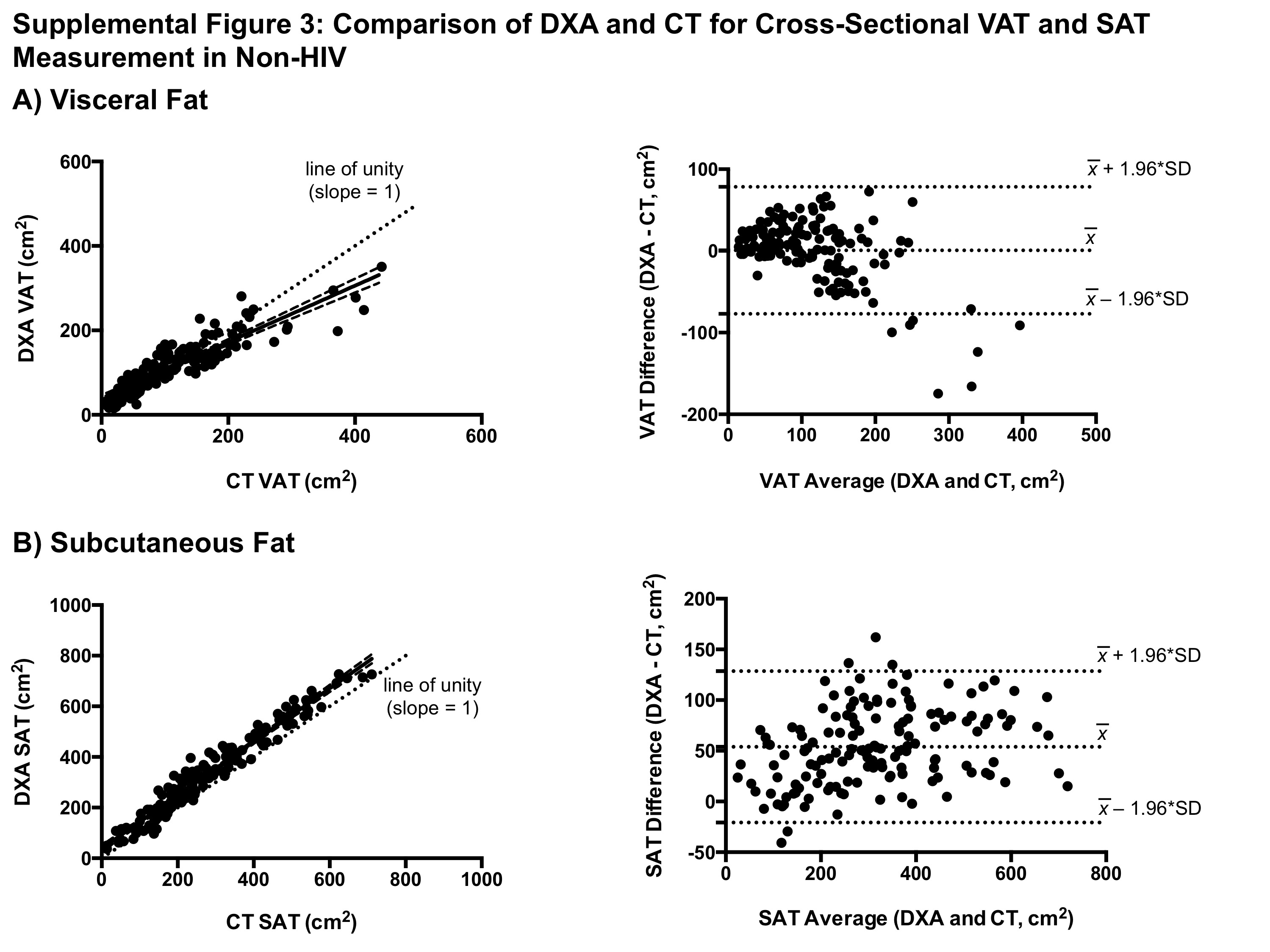

Supplement: Supplementary file 6 — Supplemental Figure 3 [file 41387_2019_73_MOESM6_ESM.jpg]

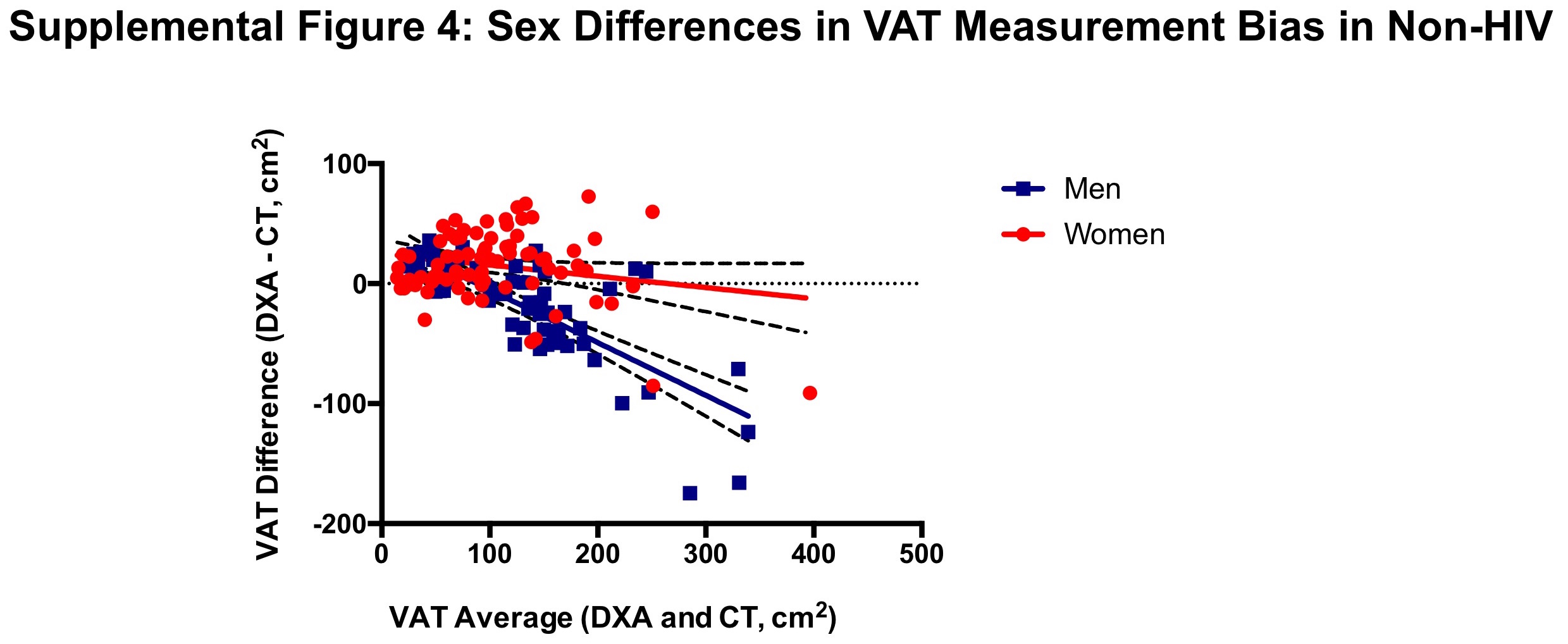

Supplement: Supplementary file 7 — Supplemental Figure 4 [file 41387_2019_73_MOESM7_ESM.jpg]

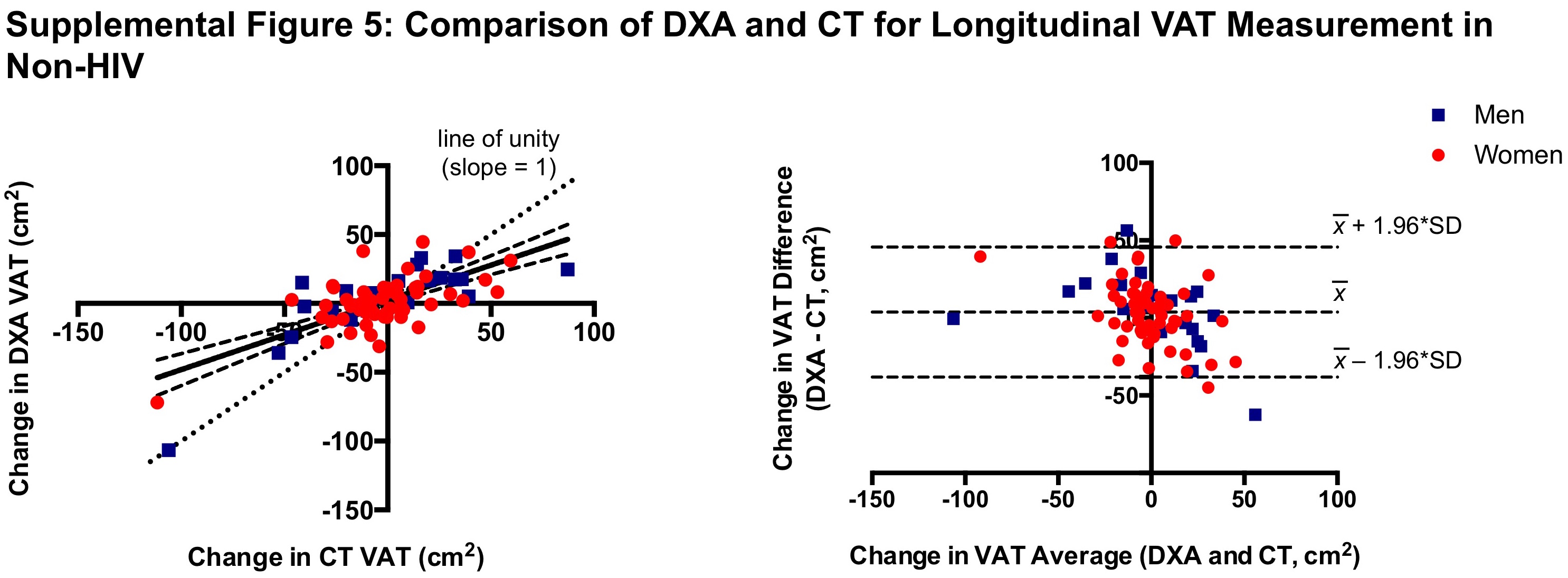

Supplement: Supplementary file 8 — Supplemental Figure 5 [file 41387_2019_73_MOESM8_ESM.jpg]
